# Timing for Pacing after Acquired Conduction Disease in the Setting of Endocarditis

**DOI:** 10.1155/2015/471046

**Published:** 2015-01-06

**Authors:** Daniel Brancheau, George Degheim, Christian Machado

**Affiliations:** Department of Cardiology, Providence Hospital and Medical Centers, 16001 West Nine Mile Road, Southfield, MI 48075, USA

## Abstract

A 53-year-old gentleman with a history of a mechanical aortic valve presented to the emergency department complaining of a sudden right-sided abdominal pain. He was found to have atrioventricular dissociation on his initial electrocardiogram and his blood cultures grew Streptococcus viridans. The suspicion for endocarditis with periaortic abscess was high so a transthoracic echocardiogram was performed and showed a mass in the left ventricular outflow tract. For better visualization, a transesophageal echocardiogram was recommended and revealed a bileaflet mechanical aortic valve with perivalvular abscess and valvular vegetation as well as severe eccentric paravalvular aortic regurgitation. After sterilization, the patient underwent a successful surgery. Postoperatively, he remained in complete heart block and a permanent pacemaker placement was performed after complete sterilization. He tolerated the procedure well and was discharged home in a stable condition. Perivalvular abscess is one of the most common cardiac complications of infective endocarditis and is associated with an increased risk of mortality. It is imperative to have appropriate treatment guidelines established. However, because of the relative nature of the disease process and the acuity at which intervention needs to be done, a true assessment of the duration of antibiotic therapy prior to surgical intervention, timing of pacemaker placement, and the type of pacemaker is controversial.

## 1. Introduction

Perivalvular abscess with high degree atrioventricular (AV) node dysfunction is one of the most common cardiac complications of infective endocarditis with a reported incidence of 30 to 40 percent. It is also associated with an increased risk of mortality. We report a case of successfully treated perivalvular abscess with complete heart block after delaying surgery and timing of permanent pacemaker placement in order for sterilization to be obtained.

## 2. Case Report

A 53-year-old gentleman presented to the emergency department complaining of a sudden right-sided abdominal pain for one day prior to admission. He also had general fatigue, myalgia, and weight loss of 10 pounds over a period of several months. His past medical history was significant for a mechanical aortic valve that had been replaced 2 years prior to hospital admission. The patient had been compliant with his warfarin; however, his presenting international normalized ratio (INR) level was 6.4.

Physical examination on admission revealed a blood pressure of 154/74, borderline bradycardia, and tachypnea. He had a mechanical S2, a 3/6 systolic ejection murmur at the apex radiating to the axilla, and a 1/4 diastolic murmur at the left lower sternal border. Other than severe tenderness in the right lower quadrant, the rest of his physical examination was essentially benign. Of note, the patient did not have any rashes, subconjunctival hemorrhage, Janeway lesions, or Roth spots.

Pertinent laboratory data revealed hemoglobin of 7.9 gm/dL and white blood cell count of 19.8 K/mcL. Computed tomography (CT) scan of the abdomen without contrast showed a large right perirenal hemorrhage extending caudally through the retroperitoneum to the right inguinal canal with evidence of active hemorrhage. Urology and general surgery evaluated the patient and were both suspecting underlying renal malignancy because visualization was obstructed from the active hemorrhage.

**Figure 1:**
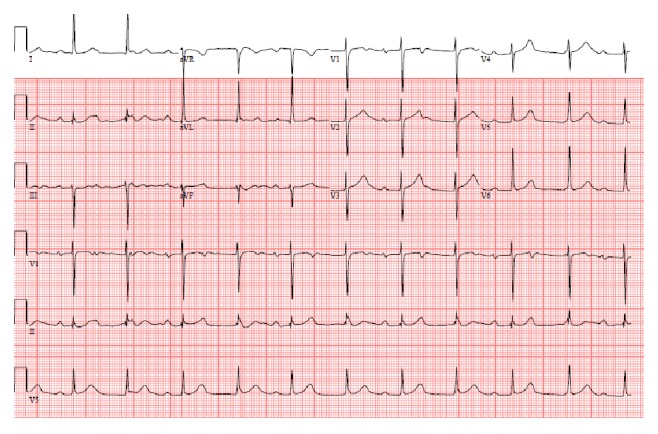
The initial electrocardiogram showing atrioventricular dissociation.

**Figure 2:**
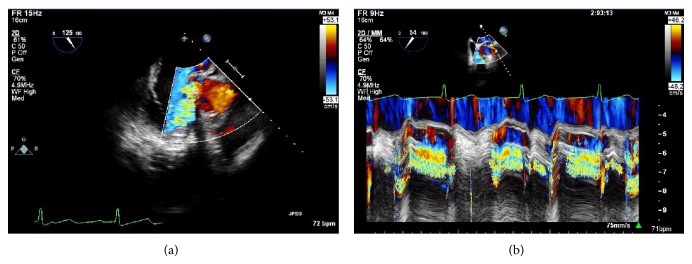
A transesophageal echocardiogram showing a bileaflet mechanical aortic valve with perivalvular abscess and valvular vegetation as well as severe eccentric paravalvular aortic regurgitation.

Cardiology was consulted because the patient had atrioventricular dissociation on his initial electrocardiogram (Figure 1). The patient was started on antibiotics and blood cultures were drawn because he met criteria for systemic inflammatory response syndrome (SIRS). The blood cultures grew* Streptococcus viridans* in six of the six bottles. With the patient's history of mechanical aortic valve, presumed embolic phenomenon with the renal hemorrhage, leukocytosis, and new third degree heart block the suspicion for endocarditis with periaortic abscess was high. Therefore, transthoracic echocardiogram (TTE) was performed which showed a mass that was present in the left ventricular outflow tract and transesophageal echocardiogram (TEE) was recommended for better visualization. The patient had worsening tachypnea and his oxygen requirements were increasing requiring the patient to be prophylactically intubated in anticipation of the fact that he would require surgical intervention in the near future. TEE was then performed and revealed a bileaflet mechanical aortic valve with perivalvular abscess and valvular vegetation as well as severe eccentric paravalvular aortic regurgitation (Figures 2(a) and 2(b)).

The patient's surgery was scheduled but not performed until the patient had negative blood cultures with antibiotic therapy because he was relatively hemodynamically stable on the ventilator. After sterilization the patient underwent a successful redo-median sternotomy, aortic root reconstruction, and aortic valve replacement.

Postoperatively, the patient remained in complete heart block but permanent pacemaker placement was withheld until completion of several more days of antibiotic therapy could be performed. After complete sterilization, permanent pacemaker placement was performed and he tolerated the procedure well with no major postoperative complications. He was later discharged home and continues to do well.

## 3. Discussion

It has been shown that surgery is superior to medical therapy in the setting of native valve complicated endocarditis [1]. It has also been shown that, in aortic and prosthetic valve endocarditis, a combination of medical and surgical treatment improves outcomes as well [2]. Performing surgical intervention in a patient with infective endocarditis complicated by paravalvular abscess is recommended and is considered a class I indication by American Heart Association (AHA) guidelines. However, there are still several things that are controversial: how long antibiotic therapy should be given prior to surgical intervention, the timing of permanent pacemaker, and whether or not a biventricular pacemaker should be implanted.

In one case series the mortality rate for patients that underwent surgery for periaortic abscess was between thirty and forty percent [3]. With the risk of mortality in patients with perivalvular abscess and high degree AV block being so high it is difficult to assess what may be the best treatment strategy in these patients. As aforementioned, there are several items of interest in these patients that need to be addressed: length of antibiotic prior to and timing of surgery, timing of pacemaker placement, and type of pacemaker to implant.

Our patient was given antibiotic therapy until sterilization of his blood cultures prior to surgical intervention. The patient remained relatively stable with regard to his hemodynamic and respiratory status after being placed on mechanical ventilator support. To improve the chances that his new implanted valve did not become infected it was decided to delay surgery as long as possible. Subsequently, the patient developed an intermittent left bundle branch block on telemetry monitoring, a definitive sign of abscess extension, and therefore the patient was taken to surgery at that time.

Postoperatively, from redo-median sternotomy, our patient remained in complete heart block but was hemodynamically stable. Since there was a concern for infection, dual chamber pacemaker placement was withheld several more days postoperatively so that antibiotic therapy could be extended. On day five postoperatively, the patient underwent placement of a dual chamber pacemaker without incident. While it is indicated to place a pacemaker for complete heart block, the timing of placement for this patient is controversial. There are case reports of other patients with complicated infective endocarditis requiring pacemaker placement but timing seems to be performed with sternotomy or not addressed [4]. Device infection is of great concern for patients with active systemic infection and timing of the pacemaker may not be best at the operative setting, especially if the patient is hemodynamically able to tolerate their arrhythmia. Our patient demonstrates that delay of pacemaker implantation may be feasible, safe, and a better treatment strategy than placing a permanent device at the time of open surgical intervention. At this time there are no guidelines as to timing of pacemaker implant and each case should be considered on an individual basis.

In our patient a dual chamber pacemaker was placed and not a biventricular pacemaker. There are two reasons for this treatment strategy. First, the patient had active infection and even though he underwent debridement and antibiotic treatment it was felt that it is better for the patient to place less needed hardware. And secondly, the biventricular pacing for atrioventricular block and systolic dysfunction (BLOCK HF) trial results had not been released when this patient presented to our institution. With the release of these results it may be argued that if he presented now in the absence of positive blood cultures biventricular pacing may be indicated [5].

With the high associated mortality rates of patients with complicated infected endocarditis it is imperative to have appropriate treatment guidelines established. However, because of the relative nature of the disease process and the acuity at which intervention needs to be done, a true assessment of the duration of antibiotic therapy prior to surgical intervention, timing of pacemaker placement, and the type of pacemaker is controversial. In our patient we showed the possible benefit of delaying surgery as long as possible in order for sterilization to be obtained. Then following redo sternotomy, delaying permanent pacemaker implantation may also be beneficial. Also, in the patient with active bleeding it is critical to delay surgery as long as possible because of the large amounts of anticoagulation given to patients in order to initiate cardiopulmonary bypass. In patients with intracranial hemorrhage it is recommended to delay surgery for 21 days if at all possible [6]. While our patient did not have an intracranial hemorrhage, he did have an extensive subcapsular renal hemorrhage that made it difficult to determine the best timing for surgical intervention. In this case, the deterioration of our patient's conduction system with a progressing left bundle branch block indicated an extension of his abscess. With his hemoglobin counts remaining stable after transfusion and his blood cultures negative for growth it was determined to be appropriate timing for fear of rupture of his aortic root.

## 4. Conclusion

While our case is not particularly rare, it does address several important points when treating complicated infective endocarditis. First, it may be safe and beneficial to delay surgical intervention as long as possible in order to give an adequate dose of antibiotics. Second, placement of a permanent pacemaker in someone with AV dissociation who is undergoing treatment for periaortic abscess may also be safe and beneficial if they can hemodynamically tolerate it. Third, dual chamber pacemaker placement versus biventricular pacing for high degree heart block in patients with periaortic abscess is controversial and not well established and probably will never be studied in a randomized controlled fashion because of the nature of the disease process. It is imperative that, in these settings, each patient should be considered on an individual basis to help providing the best clinical outcome.

## Learning Objectives

Perivalvular abscess with high grade atrioventricular nodal conduction disease is one of the most common cardiac complications of infective endocarditis. With the high associated mortality rates, it is imperative to have appropriate treatment guidelines established. There are several aspects to consider with regard to timing and management of these critical cases. First of all, it is imperative to note that surgical intervention is superior to medical therapy. Second, the timing of surgical intervention is controversial. Third, the decision on timing of permanent pacemaker and whether or not biventricular pacing should be placed is also a decision that needs to be evaluated on an individual patient basis.
